# Mid-infrared Laser Spectroscopy of Jet-Cooled Formic Acid Trimer: Mode-Dependent Line Broadening in the C–O Stretching Region

**DOI:** 10.1021/acs.jpclett.3c01860

**Published:** 2023-08-24

**Authors:** Arman Nejad, Xiang Li, Tianxin Zhu, Yun Liu, Chuanxi Duan

**Affiliations:** † Institute of Physical Chemistry, Georg-August University of Göttingen, Tammannstraße 6, Göttingen 37077, Germany; ‡ College of Physical Science and Technology, Central China Normal University, Wuhan 430079, China

## Abstract

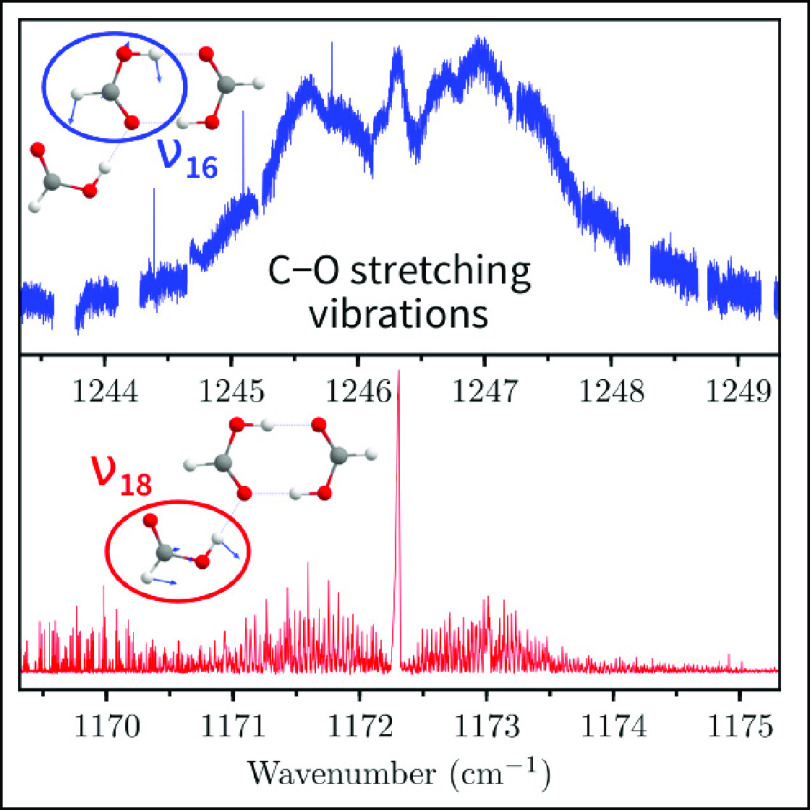

Building on recent progress in the vibrational spectroscopy of the formic acid trimer, we present the first high-resolution measurements of the jet-cooled laser absorption spectrum of (HCOOH)_3_. The spectra of the lowest- and highest-frequency C–O stretching fundamentals are analyzed whereas the third band is not observed, complicated by monomer and dimer absorptions at 1219 cm^–1^ (8.2 μm). Vibration–rotation parameters are obtained for the band at 1172.31512(68) cm^–1^ whereas the C–O stretch at 1246.33(5) cm^–1^ exhibits a significantly larger breadth, allowing only resolution of the coarse *PQR* structure. Vibrational predissociation can be ruled out, and intramolecular vibrational redistribution mechanisms are discussed, particularly coupling to the concerted proton exchange within the cyclic dimer subunit. Ultimately, the question remains open. The prospects of high-resolution measurements of other trimer bands or isotope substitution experiments, which might assist in revealing the mode-specificity of the underlying broadening mechanisms, are discussed.

Molecular vibrational spectroscopy provides important insights into the internal dynamics of molecules and clusters while also serving as an invaluable tool to rigorously test and evaluate quantum chemical approximations.^1^ One molecule that has extensively been studied is formic acid [HCOOH]. Its monomer, F, exhibits a large-amplitude OH torsion that is simultaneously entangled in a strong resonance polyad^2−4^ and its cyclic dimer, (FF), is a prototype of double hydrogen bonding and concerted proton transfer,^5,6^ to name a few interesting aspects. In contrast to the plethora of decades-spanning spectroscopic investigations of F and (FF), whose benchmark databases^7^ have considerably matured over the past seven years thanks to a very fruitful interplay of theory and experiment [see refs (8 and 9) and references cited therein], spectroscopic studies of the formic acid trimer are limited to a handful of more recent publications.^9−14^ Its global minimum conformer, F(FF), combines two important bonding motifs [Figure 1]—the double hydrogen-bonded (FF) motif, which is predominant in the gas and, for larger carboxylic acids, the crystalline phase,^15^ and the polar FF dimer motif which constitutes the building block of crystalline formic acid.^16^ As such, the vibrational spectroscopy of the formic acid trimer deserves more attention.

**Figure 1:**
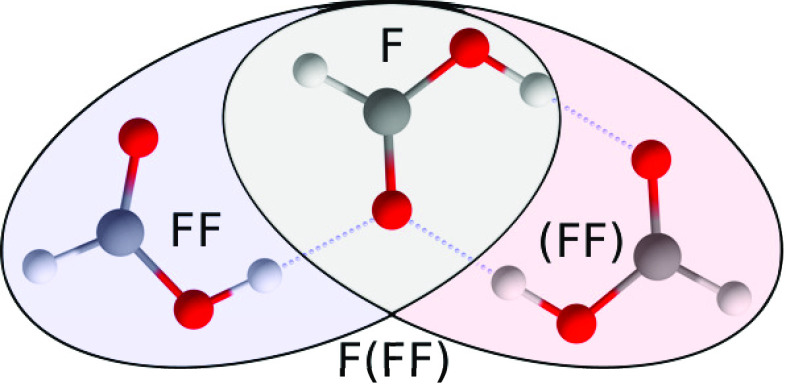
Connectivities of *trans*-formic acid monomer F, cyclic dimer (FF), polar dimer FF, and trimer F(FF). Reproduced from ref (14). Copyright 2021 AIP Publishing, licensed under a Creative Commons Attribution (CC BY) license.

We report the first rotationally resolved vibrational spectrum of the formic acid trimer by measurement of its absorption spectrum between 1100 and 1370 cm^–1^ in a supersonic expansion of HCOOH seeded in Ar. Several vibrational bands have been observed in our spectral survey, including new (FF) combination bands. In this contribution, we focus on the rotational analysis of the C–O stretching bands of F(FF) which were recently reported in jet expansions at low spectral resolution.^14^ The three C–O stretches are approximately localized on each formic acid unit, the docking of the third F thus breaking the Davydov coupling^14^ within (FF) for these particular modes. Other than the mid-frequency C–O stretch ν_17_, which is obscured by more intensive monomer and dimer absorptions, the lowest-frequency ν_18_ is spectrally well separated and its rotational fine structure fully resolved, enabling the determination of vibrationally averaged rotational and quartic distortion constants.^17^ The highest-frequency ν_16_, on the other hand, is revealed to exhibit a much larger line broadening; only its coarse-grained *PQR* structure is resolved. Below, we provide possible explanations and discuss key experiments that might elucidate the nature of these mode-specific broadening mechanisms.

The formic acid trimer was first detected in the gas phase one decade ago by means of microwave spectroscopy.^10^ Neill reported ground state rotational constants and a full set of quartic centrifugal distortion constants.^10^ F(FF), is a planar, *C*_*s*_ symmetric, near-prolate symmetric top [κ = −0.92],^10^ in agreement with earlier *ab initio* predictions by Roy and Thakkar who explored the conformational landscape of the trimer by computational means.^18^ The polar arrangement of F(FF) is consistent with earlier mass spectrometric electric field measurements^19^ that ruled out the symmetrical cyclic motif prevalent in hydrogen-bonded trimers of smaller molecules.^20−22^ The first vibrational spectroscopic detection of the trimer was achieved in 2017 in the C=O and later OH stretching range,^11,13^ utilizing linear jet-cooled Fourier-transform infrared spectroscopy [FTIR]. Previous^23,24^ matrix-isolated assignments of the trimer are predominantly believed to correspond to FF,^25,26^ the polar dimer [see also discussion in ref (11)]. A recent reanalysis of the fingerprint region revealed a multitude of new vacuum-isolated F(FF) bands in the jet-cooled spectra of HCOOH^14^ and its three deuterated isotopologues,^9^ increasing the number of low-resolution vibrational band centers from 4 to a total of 40 [including 26 deuterated ones].^9,11,13^ Its surprisingly high concentration^14^ in jet-cooled spectra can be rationalized by space-resolved FTIR imaging which suggests that F(FF) is actively formed from F and (FF) in jet expansions, both of which are abundantly available in the gas phase.^11^

The C–O stretching vibrations of formic acid are highly infrared-active,^27^ strongly mixed with the CH and OH bends,^28^ and their transition wavenumbers are very sensitive^9^ toward the isotope composition and the hydrogen bond environment in F_*n*_ clusters,^14^ making them a well-suited target for non-size-selective IR spectroscopy. Difficulties that may arise in dense spectral regions are best seen for the ν_17_ fundamental of F(FF) which is located at 1219(2) cm^–1^.^14^ In search of trimers, we reexamined our previously observed spectra in this region, which were measured with lead-salt diode lasers.^5^ As can be seen in Figure 2a, the presence of strong absorption lines corresponding to F [2ν_9_,^3^ OH torsion overtone] and (FF) [ν_22_,^5,14^ C–O stretch resonance triad] complicates the assignment of lines belonging to ν_17_ of F(FF), which we could not unambiguously identify.

**Figure 2:**
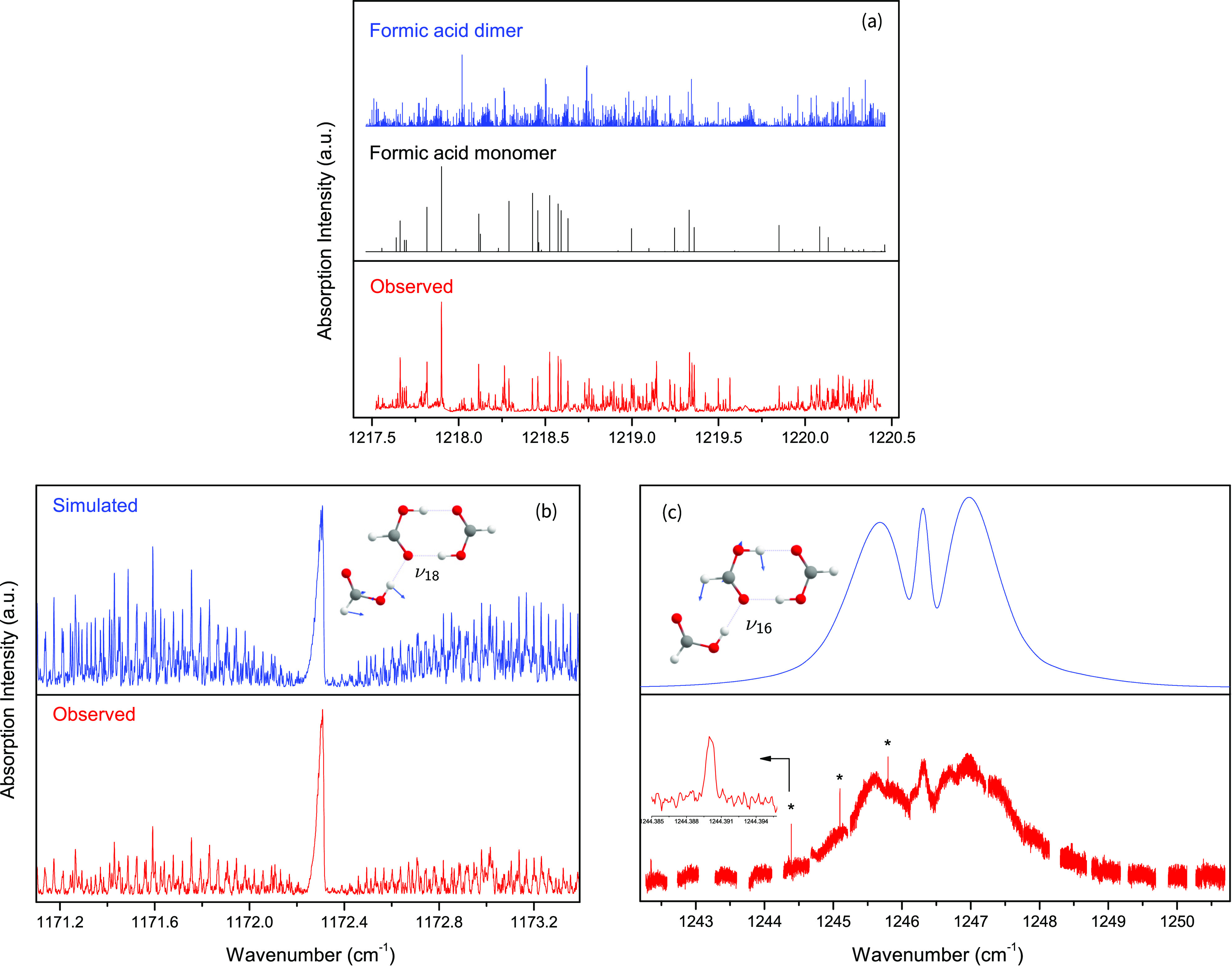
Survey of the infrared absorption spectra of HCOOH in the C–O stretching range. (a) Comparison of the observed spectrum around 1219 cm^–1^, where we expect to see ν_17_ of F(FF), with simulated spectra of F and (FF) using a rotational temperature of 12 K and a Gaussian linewidth of 0.0025 cm^–1^. (b, c) Comparison of the simulated and observed spectra of the ν_18_ and ν_16_ fundamental bands of F(FF). The normal mode displacements are shown as insets. The simulations assume a rotational temperature of 12 K and Gaussian linewidths of 0.0025 cm^–1^ in part b and 0.22 cm^–1^ in part c, respectively. Lines marked with asterisks in part c belong to the 2ν_9_ band of the formic acid monomer. An expanded view of its 6_5,1_–5_4,2_ transition with a linewidth of 0.0008 cm^–1^ is shown as an inset.

The newly measured infrared spectra of the two other C–O stretching vibrations of F(FF) are shown in Figure 2b,c. As the experimental setup has been described in detail in previous work,^29,30^ we limit the following description to a brief summary. An external-cavity quantum cascade laser [EC-QCL; Daylight Solutions, MHF-41090] with a nominated tuning range of 1090–1190 cm^–1^ was used as the tunable infrared radiation source. The piezo-actuator attached to the laser external-cavity was driven by a sine wave signal at a frequency of 50 Hz. Each scan covered a spectral range of 0.3–0.5 cm^–1^. The molecular jet was generated by supersonic expansion of HCOOH/Ar gas mixtures through a 300 μm × 80 mm slit nozzle. The HCOOH sample was kept in a stainless steel vessel at room temperature. The optimal stagnation pressure was about 3 bar. The opening time of the pulsed valves was 1.2–1.5 ms, with a repetition rate of 3 Hz. A segmented rapid-scan data acquisition method was employed.^29^ The laser beam used to probe the absorption of the molecular jet was reflected 90 times by a pair of astigmatic mirrors inside the vacuum chamber. The absorption spectrum of the ν_16_ fundamental band of F(FF) was measured using another external-cavity quantum cascade laser [Daylight Solutions, MHF-41078] with a nominated tuning range of 1240–1379 cm^–1^. The observed spectrum was calibrated by the interference fringes of a confocal Fabry–Perot interferometer [free spectral range ∼0.01 cm^–1^] and the SO_2_ or N_2_O lines listed in HITRAN.^31^

Figure 2b shows a segment of the infrared spectrum in the vicinity of the ν_18_ fundamental of F(FF). This band is an *a*/*b* hybrid vibrational band with a prominent *Q*-branch located at around 1172.3 cm^–1^. The assignment and fitting of lines were straightforward with the PGOPHER program.^32^ A total of 620 transitions were assigned in the spectral range of 1170.8–1175.4 cm^–1^ with *J* and *K*_*a*_ values up to 39 and 15, respectively. The ground state molecular constants were fixed to previous microwave values^10^ whereas the vibrational band center, the three rotational constants and four out of five quartic centrifugal distortion constants of the excited level were floated in the fit. The results are listed in Table 1. The standard deviation of the fit is small (0.00075 cm^–1^) but we note that the sizable deviations of Δ_*K*_ and δ_*K*_ to the ground state values are indicative of potential (Coriolis) resonance perturbations that would call for a separate treatment. The observed linewidths of the ν_18_ fundamental band of F(FF) are 0.0025(5) cm^–1^ and about three times larger than those of F or (FF) observed under the same experimental conditions. The rotational temperature is estimated to be 12(1) K by fitting the contour of the *Q*-branch. The *a*:*b* ratio of the transition dipole moment components is about 1.0:0.8.

**Table 1:** Molecular Constants for the ν_18_ Fundamental Band of F(FF) in Watson’s *A*-Reduced Hamiltonian in the *I*^*r*^ Representation^a^

parameter	ground state^b^	ν_18_
ν̃_0_ [cm^–1^]		1172.31512(68)
*A* [MHz]	2936.5116(4)	2930.870(70)
*B* [MHz]	595.07077(7)	593.892(10)
*C* [MHz]	495.25989(6)	494.333(11)
Δ_*J*_ [kHz]	0.07676(24)	0.111(10)
Δ_*JK*_ [kHz]	–0.2838(9)	–0.377(95)
Δ_*K*_ [kHz]	4.56(3)	0.60(35)
δ_*J*_ [kHz]	0.01674(6)	0.01674^c^
δ_*K*_ [kHz]	0.293(4)	2.93(77)

a Numbers in parentheses are one standard deviation in the unit of the last digit.

b From ref (10).

c Fixed to the ground state value.

The highest-frequency C–O stretching band, ν_16_, is located at 1246.3 cm^–1^. This wavenumber range is beyond the mode-hop-free region of the EC-QCL and close to its low-frequency limit. Therefore, there are some gaps in the observed spectrum in Figure 2c due to mode hopping. In stark contrast to the ν_18_ band, the rotational lines of ν_16_ are not limited by the laser linewidth and not resolved at the available resolution, preventing any quantitative analysis. If we assume the rotational parameters of ν_16_ are the same as those of ν_18_, we can estimate the band origin and the linewidth of ν_16_ with PGOPHER which yields 1246.33(5) cm^–1^ and 0.22(2) cm^–1^, respectively. This linewidth should be contrasted with that of ν_18_ [0.0025(5) cm^–1^, Figure 2b] and of the formic acid monomer, which has absorption lines in the same spectral region [0.0008(2) cm^–1^, Figure 2c].

Starting with the obvious explanation for the two orders of magnitude larger linewidth of the higher-frequency ν_16_, we rule out vibrational predissociation^33^ as the main cause. While no experimental value is currently available for the dissociation of F(FF) into F and (FF), we can quite reliably estimate the dissociation energy. Combining CCSD(T)-F12a/VDZ-F12 results for the electronic and harmonic zero-point vibrational components of the dissociation energy with MP2/aVTZ-quality anharmonic VPT2 corrections proved very successful recently^14^ in reproducing the experimental dissociation energy^34^ of (FF) [*D*_0_ = 59.5(5) kJ mol^–1^] within the experimental error bounds, providing a numerically robust alternative to basis set extrapolation techniques.^35^ Supplementing this high-level hybrid^36^ VPT2 protocol with realistic error bars, we expect the dissociation energy into F and FF to be within 34(2) kJ mol^–1^—more than twice the excitation energy of ν_16_. Since the linewidths of the two observed C–O stretches of F(FF) are significantly larger than those for F or (FF) under the same experimental conditions, we can further rule out broadening mechanisms such as pressure or Doppler broadening.

In the following, we address possible intramolecular vibrational redistribution (IVR) mechanisms. With several trimer conformers lying below the excitation energy of ν_16_, it is instructive to include possible isomerization pathways in the discussion. Maintaining the above-mentioned *ab initio* rigor to explore the conformational landscape of the trimer is computationally very demanding. Building on the work of Roy and Thakkar,^18^ we optimized several trimer geometries at the B3LYP-D3(BJ)^37,38^ level with Gaussian 16, Rev. A.03 [Opt = QST3 for transition states].^39^ These optimized geometries were augmented with PNO-LCCSD(T)-F12a^40^ single-point energies that we obtained with Molpro 2021.3 [DomOpt = Tight and PairOpt = Tight].^41^ Finally, B3LYP-D3(BJ) harmonic zero-point corrections were added. Detailed comparisons provided in the Supporting Information [Table S1] suggest that this protocol yields dissociation energies and relative energies within 1–2 kJ mol^–1^ relative to the above-introduced hybrid VPT2 reference.

The results of the conformer search are visualized in Figure 3. Geometries of the four lowest-energy conformers are in agreement with previous findings [note different nomenclature].^12,18^

**Figure 3:**
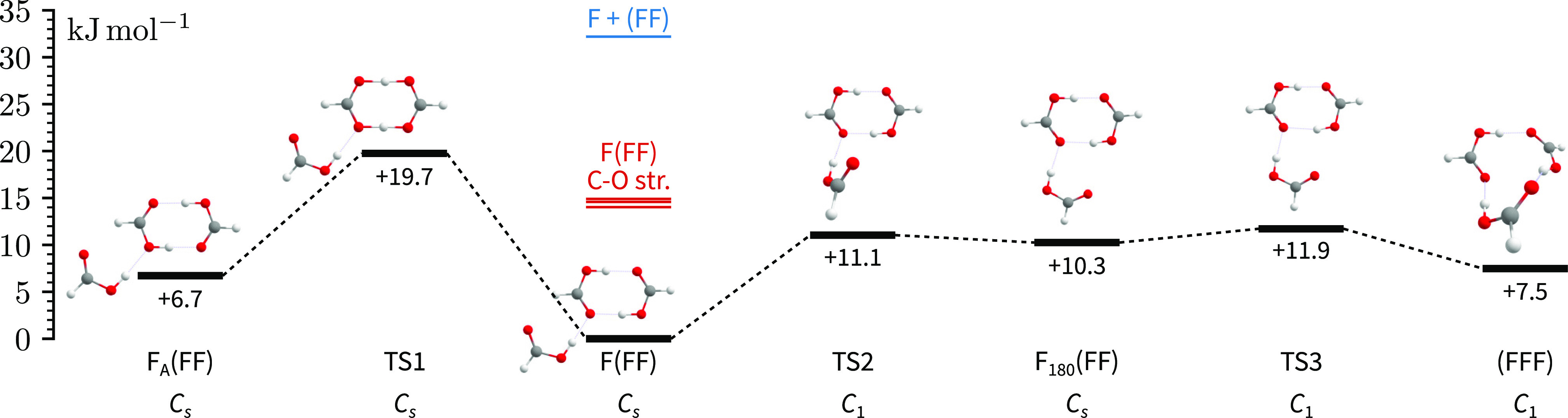
Energy profiles for the four lowest-energy formic acid trimer conformers and connecting transition states. The single-point PNO-LCCSD(T)-F12a/VDZ-F12 energies are evaluated at optimized DFT geometries, including harmonic DFT zero-point corrections [DFT ≡ B3LYP-D3(BJ)/aVTZ]. The energies required to dissociate the trimer [calculated] or excite its C–O stretching vibrations are shown in blue and red, respectively.

The first higher-energy conformer to the left of F(FF) simply differs in the phase of the concerted proton exchange coordinate in the (FF) subunit with the docking F now forming a hydrogen bond with an alcoholic oxygen. The zero-point corrected barrier is slightly increased compared to bare (FF) by about 3 kJ mol^–1^ which is almost entirely due to electronic effects. We can rule out kinetic trapping of F_A_(FF) in the jet, which should efficiently relax to the global minimum conformer. Considering the complexation dynamics,^11^ we would otherwise expect to see both conformers in the jet spectra.^14^ The impact of C–O excitation on the concerted proton exchange in the cyclic formic acid dimer is still not fully understood. In (DCOOH)_2_, excitation of the infrared-active C–O stretch was found to accelerate tunneling,^42^ whereas in (HCOOH)_2_ the tunnel-split levels are inverted compared to the ground state.^5^ It is thus conceivable that ν_16_ of F(FF) might accelerate concerted proton tunneling, making F_A_(FF) more accessible, which would lead to an increased linewidth in the spectrum. In general, excitation of (FF)-centered vibrations are expected to accelerate IVR much more than F-centered ones due to the much stronger coupling in (FF). In this case, we would expect a similar broadening in ν_17_, which unfortunately [or partly due to this?] is not observed in the spectra shown in Figure 2a.

The two remaining conformers to the right of F(FF) are connected to it through relatively low barriers of about 10 kJ mol^–1^, involving first the breaking of the weaker C=O···H–C contact between F and (FF), to allow F to rotate more freely about the O–H···O=C bond, and finally leading to ring insertion. We note that whether at low temperatures the ring is distorted^12^ or *C*_3*h*_ symmetric,^18^ sensitively depends on the electronic structure level and inclusion of vibrational anharmonicity. Figure 3 further illustrates that the excitation energy of either of the C–O stretching modes would suffice to break this contact between F and (FF). Normal mode analysis suggests that excitation of ν_16_ might lead to cleavage of the C=O···H–C contact between F and (FF) which might account for the observed mode-selective linewidths. The normal mode vectors of ν_16_ and ν_18_ are shown as insets in Figure 2b,c. Recalling that the three C–O oscillators are mostly localized on each formic acid unit, the excitation energy of ν_18_ is expected to be quickly distributed among the F vibrations and the intermolecular hydrogen bond vibrations connecting F and (FF) via the O–H··O=C hydrogen bond. The mode in question, ν_16_, on the other hand, pulls on the much weaker C=O···H–C contact. Indeed, ν_16_ involves substantial CH bending character [cf. ref (28).] which corresponds to elongation or stretching of this intermolecular “bond”.

To investigate the mode couplings in the frequency domain more quantitatively, one has to set up an effective Hamiltonian including relevant anharmonic couplings to ν_16_ and diagonalize it. Initial effective VPT2+K^43^ Hamiltonian calculations with hundreds of vibrational states were feasible but the results not very insightful. The underlying rectilinear normal coordinates are not suitable to describe highly excited van der Waals combination states that dominate the manifold nearby ν_16_. Curvilinear perturbative approaches,^44,45^ therefore, appear more promising.

To inform future theoretical studies on the formic acid trimer—or the deprotonated formic acid trimer which seems to exhibit strong anharmonic perturbations in this spectral region^46^—it is instructive to compare experimental vibration–rotation parameters of F(FF) with *ab initio* predictions. The performance of the rigid-rotor-harmonic-oscillator [RRHO] and second-order vibrational perturbation theory [VPT2] models for selected electronic structure levels and basis sets are visualized in Figure 4. While the suitability of VPT2 to describe low vibrational excitations of (FF) and F(FF) below the enigmatic OH stretching region has already been established,^9,14,35^ the lower panel of Figure 4 underscores the importance of choosing a high-level electronic structure method that is reasonably well converged with respect to the one-particle basis to calculate the harmonic force field. The performance for the rotational constants reinforces this conclusion. There are several high-level recipes for obtaining accurate rotational constants [see, for example, refs (47−49).]. A full coupled-cluster treatment, however, is computationally very expensive and practically out of reach for systems with even more, particularly non-hydrogen atoms. Figure 4 illustrates that “cheap” CCSD(T)-F12//DFT or MP2 hybrid force fields consistently provide an accuracy of 1% and below. It should be realized that the overall computational cost of the hybrid force field VPT2 is dominated by the CCSD(T)-F12 harmonic force field calculation. As such the distinguishable cluster with singles and doubles [DCSD] approach,^50^ which appears to be a very promising compromise for near-CCSD(T)-quality geometries^51^ and harmonic frequencies^52^ at the reduced cost of CCSD, warrants further investigations. Quartic centrifugal distortion constants, which can be calculated to lowest-order with VPT2,^17,49^ are reported in the Supporting Information [Table S3]. For these, we observe only a qualitative agreement with experiment—the signs and orders of magnitude are correctly predicted for the vibrational ground state, but absolute deviations amount up to 24%.

**Figure 4:**
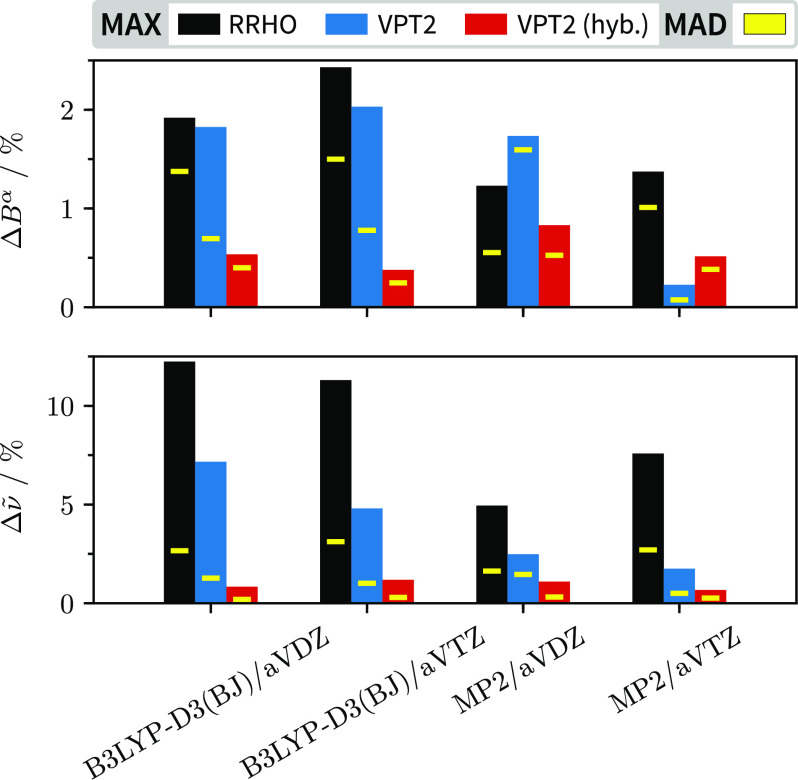
Relative absolute errors to experiment for six rotational [α denotes a principal axis] and twelve vibrational parameters of F(FF), comprising the ground state and *v*_18_ = 1 rotational constants and fundamental band centers from 270 up to 1755 cm^–1^. The absolute values are deposited in the Supporting Information [Table S2]. In the substituted hybrid approach, all harmonic force field parameters are replaced with equilibrium values at the CCSD(T)-F12a/VDZ-F12 level using only the cubic and quartic force constants from B3LYP or MP2.

We presented in this contribution the high-resolution infrared spectra of the ν_16_ and ν_18_ C–O stretching vibrations of the formic acid trimer F(FF). These two bands were found to exhibit mode-specific line broadening. Vibrational predissociation could be ruled out as the main contributor. We discussed possible mechanisms that might account for the two orders of magnitude larger linewidth of ν_16_. Future high-resolution measurements of other spectral regions or deuterated isotopologs are expected to provide important puzzle pieces to elucidate the underlying mechanism(s). If the broadening is mainly due to the increased density of ro-vibrational states, reduction of the excitation energy through costly ^18^O substitution might lead to a reduced line broadening. If, on the other hand, the broadening is not unique to ν_16_ but generally reflects an increased IVR for the more strongly bound (FF) vibrations, we should expect similar trends in other spectral regions. Particularly promising [see Figure S1 in the Supporting Information] are high-resolution measurements of the OCO bending [672 and 717 cm^–1^] and C=O stretching [1619 cm^–1^] modes which are well separated from F and (FF) vibrations and have been observed in previous jet measurements.^11,14^ If other (FF)-centered vibrations below 10 kJ mol^–1^ do not exhibit a similar broadening, this might be indicative of a ν_16_-specific mechanism possibly leading to breaking of the C=O···H–C contact between F and (FF) [see Figure 1], quite similar to previously reported ring-opening IVR of the DF stretching in (DF)_3_ where deuteration shifts the intramolecular stretch below the predissociative threshold.^21^ We were not able to identify the third and last C–O stretch ν_17_ which is obscured by nearby monomeric [OH torsion overtone] and dimeric [member of the C–O stretch resonance triad] contributions.^5^ It might similarly have an increased breadth, which would complicate its assignment. Measurement of the C–O stretching spectra of (DCOOH)_3_ and (HCOOD)_3_ might provide further insights. Since C–H deuteration not only detunes the C–O stretch resonance in (FF) but also shifts the nearby monomer band toward lower wavenumbers, formic-*d* acid appears to be a promising candidate for the detection of the missing ν_17_.^9^ Whereas O–H deuteration further reduces the shift of ν_17_ to nearby dimer bands and might complicate the assignment, it might still prove more successful should ν_17_ be broadened due to coupling with the double proton exchange. Similarly, if the line broadening of ν_16_ reduces upon O–H deuteration, the underlying mechanism might be associated with coupling to the proton transfer, accelerating tunneling to F_A_(FF) [see Figure 3]. In contrast to many experimentally studied hydrogen-bonded trimers,^20−22^ F(FF) supports several bound intramolecular vibrational levels. The cyclic ring motif, which is usually the global minimum conformation in hydrogen-bonded trimers,^20−22^ is a higher-energy form of the formic acid trimer. We encourage future high-resolution measurements of this interesting molecular complex which, due to its strong hydrogen bonding and sheer size, is furthermore an excellent reference system to benchmark quantum electronic and nuclear vibrational methods. Lastly, we would like to point out that the first reported high-resolution spectrum of (FF), i.e., the C=O stretch at 1741 cm^–1^, remains unassigned to this date.^53^ A reanalysis including the nearby trimer modes appears timely in light of recent developments.^9,11^

## Supporting Information Available

The following files are available free of charge. The Supporting Information is available free of charge at https://pubs.acs.org/doi/10.1021/acs.jpclett.3c01860.

Data supporting composite PNO/DFT single-point calculations, raw data for Figure 4, calculated centrifugal distortortion constants, and calculated infrared band strengths of other F(FF) vibrations (PDF)
