# Supplementary material for: Mid-infrared Laser Spectroscopy of Jet-Cooled Formic Acid Trimer: Mode-Dependent Line Broadening in the C–O Stretching Region
Source: J Phys Chem Lett. 2023 Aug 24;14(35):7795–801. doi: 10.1021/acs.jpclett.3c01860 (PMC10786437; doi:10.1021/acs.jpclett.3c01860)
Supplement: Supplementary file 1 — jz3c01860_si_001.pdf [file jz3c01860_si_001.pdf]

Supporting Information:

Mid-infrared Laser Spectroscopy of Jet-Cooled Formic Acid Trimer: Mode-Dependent Line Broadening in the C – O Stretching Region

Arman Nejad,[a,b*] Xiang Li,[c] Tianxin Zhu,[c] Yun Liu[c] and Chuanxi Duan[c*]

[a] *Institute of Physical Chemistry, Georg-August University of Göttingen, Tammannstraße 6, 37077 Göttingen, Germany.*
[b] *Current address: Physical and Theoretical Chemistry Laboratory, University of Oxford, South Parks Road, Oxford OX1 3QZ, UK.*
[c] *College of Physical Science and Technology, Central China Normal University, Wuhan 430079, China.*
E-mail: arman.nejad@chem.ox.ac.uk and duanchx@mail.ccnu.edu.cn

Contents

S1 Tables **S1**

S2 Figures **S3**

References **S3**

S1 Tables

Table S1 Assessment of different 'recipes' to obtain zero-point corrected dissociation energies of (FF) and F(FF). All values are in kJ mol^{-1}. The much cheaper recipes B and C, where the PNO single-point energies are evaluated at equilibrium DFT geometries and anharmonic corrections are neglected, provide dissociation energies that agree with recipe A within 2 kJ mol^{-1}. For the dissociation of (FF), all three reproduce the experimental value.[1] As such, recipe C provides an excellent trade-off between accuracy and computational cost to explore the conformational landscape of the formic acid trimer.

				(FF) → F+F			F(FF) → F+(FF)	
Electronic structure	Basis set	Geometry	D_e	ΔZPVE$_h$	ΔZPVE$_{\Delta\text{VPT2}}$	D_e	ΔZPVE$_h$	ΔZPVE$_{\Delta\text{VPT2}}$
1 B3LYP-D3(BJ)	aVDZ	eq.	76.1	−7.8	0.6	41.9	−5.6	0.3
2 B3LYP-D3(BJ)	aVTZ	eq.	74.3	−7.7	0.8	40.4	−5.6	0.4
3 MP2	aVDZ	eq.	68.7	−8.5	0.5	40.2	−5.5	0.4
4 MP2	aVTZ	eq.	70.2	−8.3	0.6	39.7	−5.7	0.5
5 PNO-LCCSD(T)-F12a	VDZ-F12	B3LYP-D3(BJ)/aVTZ	67.9	-	-	37.8	-	-
6 PNO-LCCSD(T)-F12b	VDZ-F12	B3LYP-D3(BJ)/aVTZ	67.4	-	-	37.4	-	-
7 CCSD(T)-F12a	VDZ-F12	eq.	67.7	−8.4	-	38.5	−5.3	-
Recipe A: $D_0 = D_e(7) + \Delta\text{ZPVE}_h(7) + \Delta\text{ZPVE}_{\Delta\text{VPT2}}(4)$				**60.0**			**33.8**	
Recipe B: $D_0 = D_e(6) + \Delta\text{ZPVE}_h(4)$				**59.0**			**31.8**	
Recipe C: $D_0 = D_e(6) + \Delta\text{ZPVE}_h(2)$				**59.7**			**31.8**	
Experimental D_0				**59.5(5)**			**-**	

Table S2 Rotational constants [in MHz] and vibrational band centers [in cm^{-1}] of the HCOOH trimer F(FF). These are the raw data that are used in Fig. 4 of the main text. The experimental data are from this work and the literature.[2–5] Note that some of the experimental values are rounded to one digit after the decimal point.

	Exp.	B3LYP-D3(BJ)						MP2					
		aVDZ			aVTZ			aVDZ			aVTZ		
		RRHO	VPT2	VPT2(h)	RRHO	VPT2	VPT2(h)	RRHO	VPT2	VPT2(h)	RRHO	VPT2	VPT2(h)
A_0	2936.6	2987.1	2988.3	2942.8	3002.1	2994.6	2935.4	2900.5	2895.5	2937.2	2953.8	2941.5	2930.9
B_0	595.1	601.3	594.3	591.9	600.6	594.4	592.8	593.7	584.7	590.1	602.0	594.9	592.0
C_0	495.3	500.5	496.1	493.2	500.5	496.4	493.6	492.8	486.9	491.8	500.1	495.3	492.9
A_{18}	2930.9	2987.1	2984.4	2938.9	3002.1	2990.4	2931.2	2900.5	2891.5	2933.2	2953.8	2937.5	2926.8
B_{18}	593.9	601.3	593.2	590.8	600.6	593.3	591.7	593.7	583.7	589.1	602.0	593.8	590.9
C_{18}	494.3	500.5	495.1	492.2	500.5	495.4	492.6	492.8	486.0	490.9	500.1	494.3	492.0
ν_7	1755	1775.3	1739.8	1754.5	1782.2	1750.1	1758.2	1756.1	1721.8	1756.0	1777.2	1742.9	1755.0
ν_9	1619	1649.7	1596.3	1624.2	1653.9	1596.2	1619.3	1659.7	1613.2	1634.7	1672.5	1622.2	1629.9
ν_{11}	1407	1453.5	1402.6	1406.7	1454.8	1401.8	1404.9	1450.3	1397.7	1405.8	1456.3	1405.0	1405.9
ν_{13}	1397	1407.3	1377.4	1399.2	1423.2	1385.8	1389.5	1404.8	1378.2	1401.2	1424.2	1394.8	1396.5
ν_{15}	1347	1369.1	1339.1	1358.3	1382.7	1344.4	1350.8	1368.3	1330.7	1352.2	1383.4	1340.0	1345.7
ν_{16}	1246.3	1279.1	1256.0	1250.1	1283.2	1255.0	1245.2	1261.8	1231.7	1245.5	1283.7	1255.5	1243.9
ν_{17}	1219	1254.2	1228.0	1221.0	1255.6	1226.1	1218.1	1234.1	1204.8	1218.3	1257.5	1226.2	1216.6
ν_{18}	1172.3	1202.4	1167.6	1170.8	1202.8	1169.9	1172.8	1188.1	1153.6	1171.5	1210.1	1172.4	1168.3
ν_{19}	717	729.1	722.4	717.1	735.1	731.2	720.0	712.7	703.2	714.5	725.2	716.1	714.9
ν_{20}	685	688.6	682.6	685.2	696.3	689.5	684.6	676.4	669.3	684.3	690.2	682.7	683.9
ν_{21}	672	676.3	668.5	671.5	683.8	672.9	668.6	664.5	655.3	670.4	677.7	667.0	668.9
ν_{22}	270	303.1	289.4	270.7	300.5	283.0	266.8	283.4	266.2	267.0	290.5	274.7	268.4

Table S3 Comparison of experimental[2] ground state and calculated equilibrium centrifugal distortion constants [in kHz; Watson's A-reduced Hamiltonian in I^r representation]. Note that the quartic centrifugal distortion constants do not include state-specific vibrational corrections at the VPT2 level. The overall similarity across different electronic structure levels and basis sets suggests that deviations to experiment arise from missing state-specific corrections and corrections beyond fc-CCSD(T).

	VPT2					Exp.
	B3LYP-D3(BJ)		MP2		CCSD(T)-F12a	
	aVDZ	aVTZ	aVDZ	aVTZ	VDZ-F12	
Δ_J	0.063	0.063	0.069	0.068	0.066	0.07676(24)
Δ_{JK}	−0.22	−0.22	−0.27	−0.25	−0.24	−0.2838(9)
Δ_K	3.6	3.8	3.9	4.0	3.8	4.56(3)
δ_J	0.014	0.014	0.016	0.015	0.015	0.01674(6)
δ_K	0.23	0.24	0.25	0.25	0.24	0.293(4)

S2 Figures

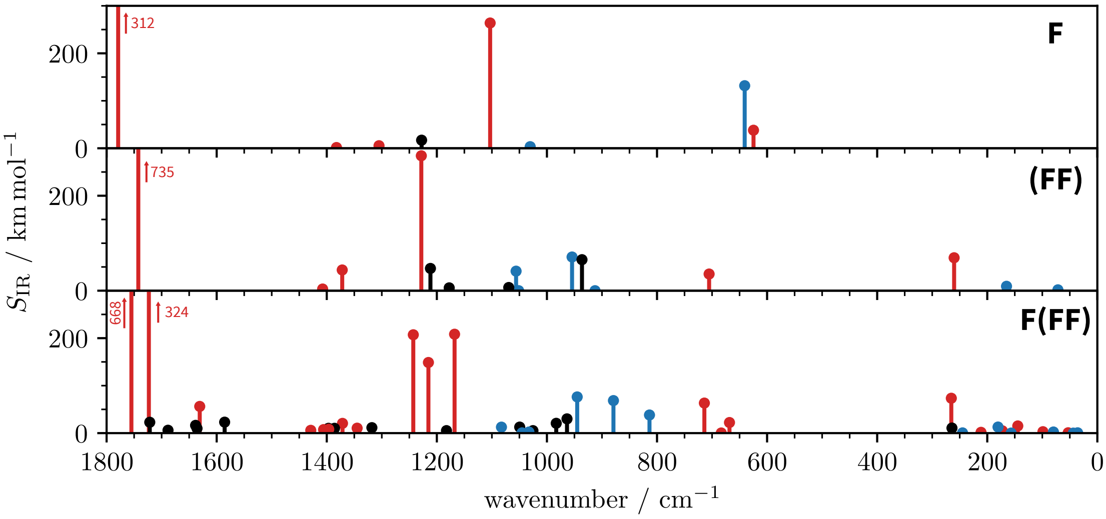

Fig. S1 Calculated VPT2 infrared spectra of monomeric [C_s], dimeric [C_{2h}], and trimeric formic acid [C_s]. In-plane [A′, B_u; red] and out-of-plane [A″, A_u; blue] fundamentals are distinguished by their color. Binary combination or overtone states with a calculated intensity above 5 km mol^{-1} are included in black. A substituted hybrid CCSD(T)-F12a/VDZ-F12//MP2/aVTZ force field was used. The data have partly been published in Ref. [6, Table VII]. Further details on the VPT2 calculations can be found there.

References

[1] F. Kollipost, R. Wugt Larsen, A. V. Domanskaya, M. Nörenberg, and M. A. Suhm, “Communication: The highest frequency hydrogen bond vibration and an experimental value for the dissociation energy of formic acid dimer”, *J. Chem. Phys.* **136**, 151101 (2012).

[2] J. L. Neill, *A new generation of chirped pulse rotational spectroscopy with applications to structure determination and astrochemistry*, doi 10.18130/V3VP1B (University of Virginia Library, Charlottesville, Virginia, 2011).

[3] K. A. E. Meyer and M. A. Suhm, “Formic acid aggregation in 2D supersonic expansions probed by FTIR imaging”, *J. Chem. Phys.* **147**, 144305 (2017).

[4] A. Nejad and E. L. Sibert III, “The Raman jet spectrum of *trans*-formic acid and its deuterated isotopologs: combining theory and experiment to extend the vibrational database”, *J. Chem. Phys.* **154**, 064301 (2021).

[5] A. Nejad, *Vibrational dynamics of formic acid and its dimer: ftir and raman jet spectroscopy and theory*, doi 10.53846/goediss-9602 (Niedersächsische Staats- und Universitätsbibliothek Göttingen, Göttingen, 2022).

[6] A. Nejad, K. A. E. Meyer, F. Kollipost, Z. Xue, and M. A. Suhm, “Slow monomer vibrations in formic acid dimer: stepping up the ladder with FTIR and Raman jet spectroscopy”, *J. Chem. Phys.* **155**, 224301 (2021).
